# Protein Glycopatterns in Bronchoalveolar Lavage Fluid as Novel Potential Biomarkers for Diagnosis of Lung Cancer

**DOI:** 10.3389/fonc.2020.568433

**Published:** 2021-01-14

**Authors:** Lina Liu, Dan Li, Jian Shu, Li Wang, Fan Zhang, Chen Zhang, Hanjie Yu, Mingwei Chen, Zheng Li, Xuan Guo

**Affiliations:** ^1^ Department of Clinical Laboratory, Xi’an No. 4 Hospital, Xi’an, China; ^2^ Department of Clinical Laboratory, The First Affiliated Hospital of Xi’an Jiaotong University, Xi’an, China; ^3^ Department of Respiratory and Critical Care Medicine, The First Affiliated Hospital of Xi’an Jiaotong University, Xi’an, China; ^4^ Laboratory for Functional Glycomics, College of Life Sciences, Northwest University, Xi’an, China

**Keywords:** lung cancer, bronchoalveolar lavage fluid, protein glycosylation, lectin microarray, diagnostic model

## Abstract

Lung cancer is one of the most prevalent and life-threatening neoplasias worldwide due to the deficiency of ideal diagnostic biomarkers. Although aberrant glycosylation has been observed in human serum and tissue, little is known about the alterations in bronchoalveolar lavage fluid (BALF) that are extremely associated with lung cancer. In this study, our aim was to systematically investigate and assess the alterations of protein glycopatterns in BALF and possibility as biomarkers for diagnosis of lung cancer. Here, lectin microarrays and blotting analysis were utilized to detect the differential expression of BALF glycoproteins from patients with 80 adenocarcinomas (ADC), 77 squamous carcinomas (SCC), 51 small cell lung cancer (SCLC), and 73 benign pulmonary diseases (BPD). These 281 specimens were then randomly divided into a training cohort and validation cohort for constructing and verifying the diagnostic models based on the glycopattern abundances. Moreover, an independent test was performed with 120 newly collected BALF samples enrolled in the double-blind cohort to further assess the clinical application potential of the diagnostic models. According to the results, there were 15 (e.g., PHA-E, EEL, and BPL) and 14 lectins (e.g., PTL-II, LCA, and SJA) that individually showed significant variations in different types and stages of lung cancer compared to BPD. Notably, the diagnostic models achieved better discriminate power in the validation cohort and exhibited high accuracies of 0.917, 0.864, 0.712, 0.671, and 0.781 in the double-blind cohort for the diagnosis of lung cancer, early stage lung cancer, ADC, SCC, and SCLC, respectively. Taken together, the present study revealed that the abnormally altered protein glycopatterns in BALF are expected to be novel potential biomarkers for the identification and early diagnosis of lung cancer, which will contribute to explain the mechanism of the development of lung cancer from the perspective of glycobiology.

## Introduction

Lung cancer is the most frequent malignancy, with the highest incidence and mortality in both sexes worldwide, accounting for approximately 2.1 million new cases (11.6% of all tumors) diagnosed and 1.8 million deaths (18.4% of all tumors) per year globally, which seriously endangers human health (1). According to the different pathological characteristics, lung cancer is classified into small cell lung cancer (SCLC) and non-SCLC (NSCLC) clinically (2). The latter can be further subdivided into three main histological subtypes of adenocarcinoma (ADC), squamous cell carcinoma (SCC), and large cell carcinoma, and rarer variants such as mixed or undifferentiated pulmonary carcinomas (2). The major cause of this poor prognosis is primarily related to diagnosis at an advanced stage (stage III or IV) for the majority of patients with lung cancer and their therapeutic limitations that miss the optimal treatment opportunity (3). Therefore, early diagnosis is pivotal, and it is estimated that 36%–73% of patients will survive longer than 5 years if they are identified at an early stage (stage I or II) (4, 5).

To improve the early detection and outcome of lung cancer patients, several systematic randomized clinical trials have recommended using high-sensitivity imaging technology such as low-dose computed tomography (LDCT) as a screening tool for monitoring high-risk individuals (6). Although LDCT scan can reduce mortality, its clinical utility is restricted due to the high false positive rate with multiple screenings and unnecessary radiation exposure (7). Furthermore, substantial progress has been made in our understanding of tumor biological processes and advancement in treatment strategies, which has led to the development of targeted therapy for lung cancer with significant improvement in the overall progression-free survival rate (8). Despite this array of new targeted immunotherapy treatments, a cure remains elusive for the majority of patients because of the inevitable drug resistance (9). Consequently, to overcome the obstacles and complement current diagnosis and screening methods, the discovery of potential protein biomarkers with high sensitivity and specificity for early detection, therapy guidance as well as prognosis monitoring is an urgent priority. To date, numerous studies have focused on searching for indicators in blood, yet the clinical application of traditional serum-based biomarkers is still far from satisfactory owing to its low diagnostic efficacy (10–12). Currently, protein biomarker detection using body fluids such as urine, saliva, exhaled breath condensate, and pleural effusion has emerged as a promising modality for cancer diagnosis and monitoring of disease progression mainly because of its minimal invasiveness and easy accessibility (13–16). Bronchoalveolar lavage fluid (BALF), a type of proximal biofluid routinely obtained from the segmental bronchus of interest during flexible fiberoptic bronchoscopy in individuals with suspected pulmonary disease, has also been considered as a useful, safe and minimally invasive biological specimen for lung cancer biomarker discovery (17). By utilizing this approach, airway components can be recovered from a large area of lung parenchyma (18). This is particularly important for preinvasive and early cancer research, as these primary lesions may have no visible histological changes under bronchoscopy or cannot be reached by the biopsy needles, which makes BALF potentially useful in the clinical for early diagnosis of lung cancer (19). Interestingly, the fraction of BALF that is not required for standard pathological procedures could be conveniently used for proteomics analysis and lung cancer biomarker detection. BALF has its merit in that it provides varied information including immunologic, inflammatory and infectious processes and can directly reflect the true physiological or pathological status of the patients (17). Detection of proteins in BALF from patients with lung cancer can provide direct information on exposure within the lung.

Glycosylation is one of the most critical and heterogeneous post-translational modifications during protein biosynthesis, and it is an enzyme-directed site-specific process, which is also critical for a wide range of biological processes, including microbial infection, cell differentiation, tumor metastasis, as well as cell carcinogenesis (20, 21). To our knowledge, more than 50% of cellular proteins, including most secreted proteins, cell surface proteins and intracellular proteins, are glycoproteins modified by different types of glycan structures that closely reflect the physiological status of the cell (22). Hence, research efforts concentrating on the effect of disease state on the glycan biosynthesis may be more direct and evident than that of cancer-related protein alterations, which also contributes to the diagnosis and understanding of disease (23). It is now well manifested that the formation of abnormal glycosylation is a key feature of malignant transformation of tumor cells (24, 25). Altered protein glycosylation often occurs early in tumor development, and the expressions of certain tumor-associated glycans in precursor lesions of different types of cancer have become powerful early diagnostic markers (23). Aberrant glycosylation has been observed during the development and progression of lung cancer, including changes in expression, fucosylation, N-glycan branching types, and increased sialylation on proteins or glycolipids (26–28). By using systemic glycomics strategies, we can further examine disease-related changes in glycoproteins. Researchers detected the differential glycopatterns of lung cancer tissue and nonmalignant tissue at the level of individual glycan structures by nLC-chip-TOF-MS (29). In addition, the relevant study also developed a serum mass profile-based signature to identify patients with early stage of lung cancer, which revealed that several components with abundances could distinguish patients with early-stage lung cancer from healthy high-risk smokers (30–32). In the past two decades, the use of lectins is one of the main methods to study glycosylation (33). A lectin microarray is composed of a group of lectins with unique glycan-binding properties printed on a solid support. These lectins are immobilized in a high-density matrix and exhibit a multivalent display (34). Currently, with the emergence of high-throughput glycomic techniques, lectin microarrays are capable of quantitative analysis of N- and O-linked glycans simultaneously based on subtle differences with minimal sample preparation and have become a primary and valuable approach for investigating the glycosylation of original intact samples without the need for glycan release, separation or purification (34, 35). Furthermore, analysis of various types of biological specimens, such as cells, tissues, and body fluids, by lectin microarrays has been developed in different diseases (36–41). For instance, Hirao et al. (42) performed lectin microarray analysis on lung cancer tissue and cell lines and found AAL, HHL, and ConA as lectin probes specific to NSCLC.

In this exploratory study, to investigate lectin-specific glycosylation changes in BALF associated with lung cancer, lectin microarrays were applied to compare different or similar alterations in glycopatterns between benign pulmonary disease (BPD) and lung cancer with different types (including ADC, SCC, and SCLC) as well as different stages [including early stage lung cancer (LC-ES) and advanced stage lung cancer (LC-AS)]. In addition, we also assessed the possibility of aberrant glycopatterns in BALF as novel potential biomarkers for the identification and early diagnosis of lung cancer.

## Materials and Methods

### Ethics Statements

The collection and use of all human BALF samples for research presented here were approved by the Ethical Committee of the First Affiliated Hospital of Xi**’**an Jiao Tong University in Xi**’**an, China. Written informed consent was received from patients for the collection of their BALF. The study methodologies were conducted in accordance with the ethical guidelines of the Declaration of Helsinki.

### Training Cohort and Validation Cohort

BALF samples were obtained from patients who were undergoing fiberoptic bronchoscopy examination at the First Affiliated Hospital of Xi’an Jiaotong University from July 2018 to March 2019. A total of 208 diagnosed lung cancer (80 ADC, 77 SCC, and 51 SCLC), 73 clinical controls with detected BPD but only with non-malignant lung disease consisting of pneumonia, tuberculosis and bronchiectasis, as confirmed by biopsy, were selected for the current study. All 281 subjects were randomly divided into a training cohort (n=163) and a validation cohort (n=118) for the construction and verification of the diagnostic models. Patients were studied in terms of their baseline clinicopathological characteristics and are presented in **Table 1**. The enrolled patients were newly diagnosed with the disease by histopathology, and those who had taken any treatment, such as preoperative radiotherapy, chemotherapy, chemoradiotherapy or curative, were excluded. BALF samples were collected by instillation and aspiration of 10 to 20 ml of sterile saline (0.9%) in the appropriate bronchopulmonary segment during fiberoptic bronchoscopy. After extraction from the respiratory airways, the majority of BALF samples appeared to be clear, and any samples with a slightly reddish appearance due to blood contamination were excluded. Approximately 10 ml of collected BALF was immediately placed on ice and thereafter Protease Cocktail Inhibitor added at a concentration of 1 μl/ml BALF to minimize protein degradation. The total volume was then centrifuged at 4,000 rpm and 4°C for 20 min to remove the cellular fraction and macromolecular insoluble materials. The supernatant was collected and then concentrated using 4 ml 3 kDa Amicon centrifugal filters. After the protein concentration was determined by the BCA assay (Beyotime Institute of Biotechnology, China), the resultant BALF was aliquoted into 1.5 ml cryotubes and stored at -80°C until the consecutive analysis.

**Table 1:** Baseline clinicopathological characteristics of the study cohorts.

Characteristics	Training Cohort				Validation Cohort				Double-blind Cohort			
	BPD	ADC	SCC	SCLC	BPD	ADC	SCC	SCLC	BPD	ADC	SCC	SCLC
n	40	48	45	30	33	32	32	21	36	31	32	21
Age, y, mean ± SD	53.4± 15.5	59.1± 11.0	64.0.± 8.9	57.0± 11.8	52.7± 15.9	60.1± 9.2	63.3± 6.8	60.4± 8.8	54.1± 13.9	55.3± 10.0	66.3± 8.6	57.3± 9.0
Sex												
Male/Female	29/11	28/20	42/3	26/4	20/13	13/19	32/0	15/6	23/13	14/17	30/2	18/3
Pathological (AJCC) ^a^												
I+II		5	9			8	14			9	6	
III+IV		34	21			19	14			18	19	
LD				10				8				12
ED				11				9				9
Unknown		9	15	9		5	4	4		4	7	0
Tumor (T)												
T1+T2		15	15	2		15	23	1		7	13	1
T3+T4		19	14	1		11	4	4		20	10	3
T_X_		3	1	0		1	0	0		0	0	0
Unknown		11	15	27		5	5	16		4	9	17
Node (N)												
N0		10	7	2		8	9	2		11	5	0
N1		3	5	0		5	6	0		3	4	0
N2		7	11	1		4	4	1		7	9	1
N3		13	7	0		8	8	2		6	5	3
N_X_		4	0	0		2	0	0		0	0	0
Unknown		11	15	27		5	5	16		4	9	17
Metastasis (M)												
M0		13	17	2		10	21	2		14	15	1
M1+M2		24	13	1		17	6	3		13	8	3
Unknown		11	15	27		5	5	16		4	9	17

^a^AJCC, American Joint Committee on Cancer staging system (7th edition); BPD, benign pulmonary disease; ADC, adenocarcinoma; SCC, squamous carcinoma; SCLC, small cell lung cancer; LD, limited stage of small cell lung cancer; ED, extensive stage of small cell lung cancer.

### Double-Blind Cohort

To properly confirm the availability of the diagnostic models established in the training cohort, another independent cohort of 120 BALF specimens was collected between April 2019 and July 2019 at the same hospital using similar selection criteria and sample processing strategies described above to serve as the double-blind cohort for this study. The results were compared with the clinical final diagnosis to evaluate the diagnostic value for lung cancer. A summary of the patient’s clinical characteristics of each group is also provided in **Table 1**.

### Fluorescent Labeling of BALF Proteins

First, 100 μg of BALF proteins were labeled with Cy3 fluorescent dye (GE Healthcare, Buckinghamshire, UK). Next, labelled proteins were separated from the excess free dye by Sephadex G-25 columns (GE Healthcare) according to the manufacturer’s instructions. Finally, the purified Cy3-labeled BALF proteins were quantified and stored at -20°C in the dark until processing.

### Lectin Microarray

**Figure 1:**
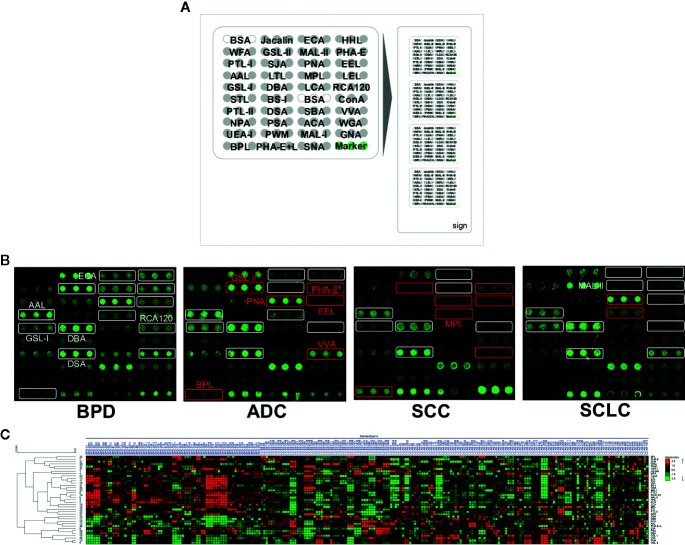
The different bronchoalveolar lavage fluid (BALF) glycopatterns in benign pulmonary diseases (BPD), adenocarcinomas (ADC), squamous carcinomas (SCC), and small cell lung cancer (SCLC) using a lectin microarray, respectively. **(A)** The layout of the lectin microarrays. Each lectin was spotted in triplicate per block with quadruplicate blocks on one slide. Cy3-labeled BSA was spotted as a location marker and unlabeled BSA as a negative control. **(B)** The binding profiles of Cy3-labeled BALF glycoproteins bound to the lectin microarrays. The lectin microarrays revealed significant signal differences among BPD, ADC, SCC, and SCLC marked with white frames. While the significant differences among three types LC marked with red frames. **(C)** Heat map and unsupervised average linkage HCA of the normalized data of 37 lectins in the 281 BALF samples. Each sample were listed in columns, and the lectins were listed in rows. The color and intensity of each square indicated expression levels relative to the other data in the row. Red, high; green, low; black, medium.

The Cy3-labeled proteins were incubated in a lectin microarray to detect different glycoproteins among clinical samples. A lectin microarray was produced using 37 lectins (Vector Laboratories, Sigma-Aldrich, and Calbiochem) with different binding preferences covering N- and O-linked glycans that were spotted on homemade epoxysilane-coated slides with Stealth micro spotting pins (SMP-10B) (TeleChem,USA) using a Capital smart microarrayer (CapitalBio Beijing, China). The specifically recognized glycan structures by lectin are summarized in the **Supplementary Material**, **Table S1**. The concentration of each lectin was 1 mg/ml in a buffer recommended by the manufacturer containing 1 mM of the appropriate monosaccharide. As shown in **Figure 1A**, each lectin was spotted in triplicate per block with quadruplicate blocks on one slide. BSA and BSA conjugated with Cy3 were used as negative and positive controls to verify the feasibility of the lectin microarray. The slides were placed in a humidity-controlled incubator at 50% humidity overnight to immobilize the lectins. After immobilization, the slides were blocked with blocking buffer containing 2% BSA in 1×PBS (0.01 mol/L phosphate buffer containing 0.15 mol/L NaCl, pH 7.4) for 1 h and rinsed twice with 1× PBS. Then, 6 μg of Cy3-labeled BALF proteins diluted in 120 μl of hybridization buffer (2%, w/v, BSA, 500 mM glycine and 0.1%, v/v, Tween-20 in PBS, pH 7.4) was incubated on the blocked slide within the chamber for 3 h at room temperature in the dark. After incubation, the microarray was rinsed twice with 1× PBST (0.2%, v/v, Tween 20 in 1× PBS, pH 7.4) for 5 min each, followed by a final rinse in 1× PBS and dried *via* centrifugation at 600 rpm for 5 min. The microarrays were scanned at 70% photomultiplier tube and 100% laser power settings using a Genepix 4000B confocal scanner. The acquired images were analyzed at 532 nm for Cy3 detection by Genepix 3.0 software.

### SDS-PAGE and Lectin Blotting Analysis

Glycosylation alterations detected by lectin microarrays between BPD controls and patients with different stages of lung cancer were further verified by SDS-PAGE and lectin blotting. To normalize the differences between subjects and to tolerate individual variation, 100 μl of each sample from BPD, LC-ES, and LC-AS were pooled. The pooled BALF proteins of each subject were analyzed by SDS-PAGE, and lectin blotting.

For SDS-PAGE, samples were mixed with 5 × loading buffer, boiled for 5 min at 100°C and then electrophoresed on a 3% polyacrylamide stacking gel and a 10% resolving gel. After electrophoresis, some gels were stained directly with silver nitrate.

For lectin blotting, the proteins in gels were then transferred onto a polyvinylidene difluoride membrane (Immobilon-P; Millipore Corp. Bedford, MA, U.S.A.) with a wet transfer unit (Hoefer Scientific) for 1.5 h at 300 mA. After transfer, the membranes were washed four times with TBS (150 mM NaCl, 10 mM Tris-HCl, 0.05% v/v Tween20, pH 7.5) and then blocked for 1 h with Carbo-Free Blocking Solution (Vector, Burlingame, CA) at room temperature. The membranes were then washed again and incubated with Cy5 (GE Healthcare, Buckinghamshire, UK)-labeled RCA120, AAL, LCA, and WFA (2 μg/ml in Carbo-Free Blocking Solution) with gentle shaking overnight at 4°C in the dark. The membranes were then washed twice each for 10 min with TTBS and scanned by a red fluorescence channel (635 nm excitation/650 LP emission) with the voltage of 800 PMT using a phosphor imager (Storm 840, Molecular Dynamics).

### Statistical Analysis

In order to minimize possible systematic variation, the median normalization method for the original lectin microarrays data was as follows. The net fluorescence intensity value of each spot was calculated by subtracting the average background value, and the values that were less than the average background ±2 standard deviations (SD) were removed from each data point. The median of the effective data points for each lectin was globally normalized to the sum of the medians of all effective data points for each lectin in a block, and we named these the normalized fluorescent intensities (NFIs). The NFI data were further analyzed by Expander 8.0 (http://acgt.cs.tau.ac.il/expander/) to perform an unsupervised average hierarchical cluster analysis (HCA).

Statistical differences between two arbitrary data sets or multiple data sets were first evaluated using a Kruskal-Wallis test, followed by a Dunn’s Multiple Comparison Test to correct for multiple comparisons through GraphPad Prism 8.0 software (GraphPad, La Jolla, CA, USA), and values of *p < 0.05, ** *p* < 0.01 or *** *p* < 0.001 were considered statistically significant. Five diagnostic models including Model LC, Model ADC, Model SCC, Model SCLC, and Model LC-ES were constructed according to the glycopattern abundances based on a forward binary stepwise logistic regression analysis using SPSS version 22.0. The discriminatory performances of candidate lectins and diagnostic models were measured using the area under the curve (AUC) on receiver operating characteristic (ROC) curve analysis by Origin 8.0 software.

## Results

### Alterations in BALF Glycopatterns Among BPD, ADC, SCC, and SCLC Detected by Lectin Microarrays

**Figure 2:**
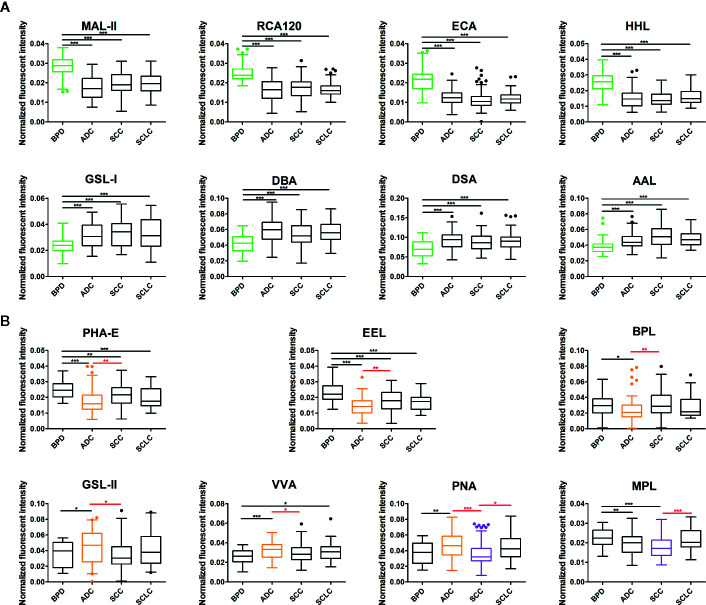
Box plot analysis of the data obtained with the 15 candidate lectins first by Kruskal-Wallis test, followed by Dunn’s Multiple Comparison Test. **(A)** The difference of bronchoalveolar lavage fluid (BALF) glycopatterns between benign pulmonary diseases (BPD) and LC (including different types) marked with black. **(B)** The difference between adenocarcinomas (ADC), squamous carcinomas (SCC), and small cell lung cancer (SCLC) marked with red. Those differences were indicated by the p-value (*p < 0.05, **p < 0.01, and ***p < 0.001).

To identify the abnormal glycopatterns associated with lung cancer, all samples from BPD, ADC, SCC, and SCLC were separately detected using lectin microarrays. The layout of the lectin microarrays and Cy3-labeled BALF glycoproteins from four subjects bound to the lectin microarrays are shown in **Figures 1A, B**. The generated data from three biological replicates of each sample were imported into Expander 8.0 software and analyzed by HCA to achieve the hierarchical relationship based on the similarities and differences among all glycopattern abundances. As shown in **Figure 1C**, the normalized data from 281 samples were distributed in the heat map. The expression levels of BALF glycoproteins among BPD, ADC, SCC, and SCLC showed obvious differences through different colours. The NFIs of each candidate lectin were further represented in a box plot by the Kruskal-Wallis test to show the variable expression levels of BALF glycopatterns. In total, 15 lectins revealed significant alterations in glycan expression among BPD, ADC, SCC, and SCLC (**Figure 2**). As shown in **Figure 2A**, the results showed that the Siaα2-3Galβ1-4Glc(NAc)/Glc binder MAL-II, the Galβ-1,4GlcNAc (type II), the Galβ1-3GlcNAc (type I) binders RCA120 and ECA, as well as the High-Mannose binders HHL, exhibited significantly decreased NFIs in all patients with lung cancer compared with BPD (all *p* < 0.001). In contrast, the αGalNAc binders GSL-I and DBA, the β-D-GlcNAc and (GlcNAcβ1-4)n binder DSA, and the Fucα1-6 GlcNAc(core fucose) binders AAL exhibited significantly increased NFIs in all patients with lung cancer compared with BPD (all *p* < 0.001). However, there was no significant difference among the three subtypes of lung cancer. Meanwhile, the lectins revealed significant differences among ADC, SCC, and SCLC, as shown in **Figure 2B**. The bisecting GlcNAc binders PHA-E, the Galα1-3(Fucα1-2)Gal (blood group B antigen) binders EEL, and the Galβ1-3GalNAc binders BPL were associated with decreased NFIs in ADC compared with BPD and SCC (all *p* < 0.05). In contrast, the GlcNAc binders GSL-II, the GalNAcα-Ser/Thr(Tn) binders VVA, and the Galβ1-3GalNAcα-Ser/Thr(T) binder PNA showed significantly increased NFIs in patients with ADC compared with BPD and SCC (all *p* < 0.05). Notably, a decrease in the NFIs of PNA was observed in SCC compared with ADC and SCLC (all *p* < 0.05). Also, the Galβ1-3GalNAc binders MPL showed a decreased in NFIs in patients with SCC compared with SCLC (*p* < 0.001).

### Alterations in BALF Glycopatterns During the Development and Progression of Lung Cancer

**Figure 3:**
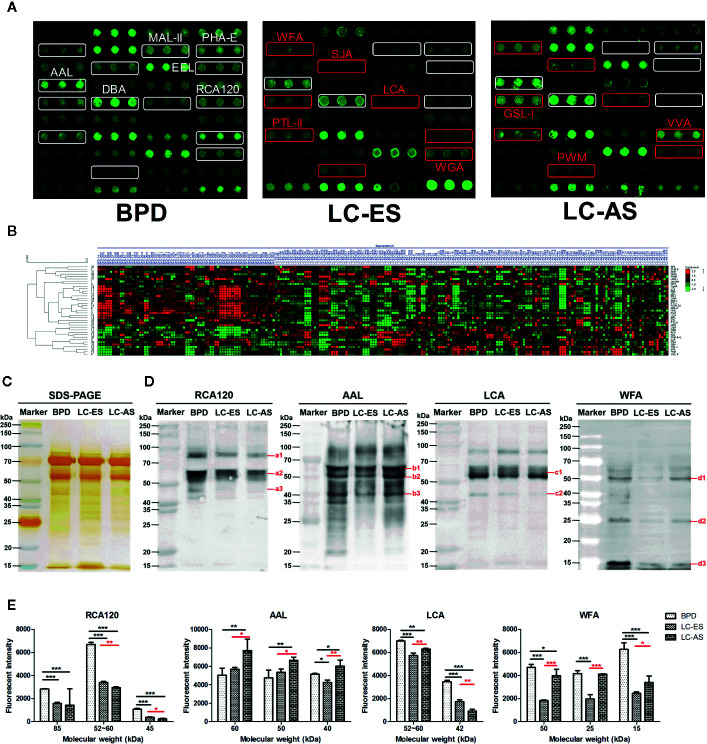
The different bronchoalveolar lavage fluid (BALF) glycopatterns in benign pulmonary diseases (BPD), early stage lung cancer (LC-ES), and advanced stage lung cancer (LC-AS) using lectin microarray and blotting analysis. **(A)** The binding profiles of Cy3-labeled BALF glycoproteins bound to the lectin microarrays. The lectin microarrays revealed significant signal differences among BPD, LC-ES, and LC-AS marked with white frames. While the significant differences among LC-ES and LC-AS marked with red frames. **(B)** Heat map and unsupervised average linkage hierarchical cluster analysis (HCA) of the normalized data of 37 lectins in the 235 BALF samples. Each sample were listed in columns, and the lectins were listed in rows. The color and intensity of each square indicated expression levels relative to the other data in the row. Red, high; green, low; black, medium. **(C)** SDS-PAGE analysis. **(D)** Binding pattern profiles of glycoproteins from BALF samples of BPD, LC-ES and LC-AS stained by four Cy5-labeled lectins (RCA120, AAL, LCA, and WFA). Blot affinity results showed 3, 3, 2, and 3 apparent bands belong to different molecular weight ranging from 15 to 250kDa, which were marked as a1-a3, b1-b3, c1-c2, and d1-d3 bound by RCA120, AAL, LCA, and WFA, respectively. a1, 85kDa; a2, 52~60kDa; a3, 45kDa; b1, 60kDa; b2, 50kDa; b3, 40kDa; c1, 52~60kDa; c2, 42kDa; d1, 50kDa; d2, 25kDa; d3, 15kDa. **(E)** The fluorescent intensities of each band with apparent difference were read by Image J. Those differences were indicated by the p-value (*p < 0.05, **p < 0.01, and ***p < 0.001).

**Figure 4:**
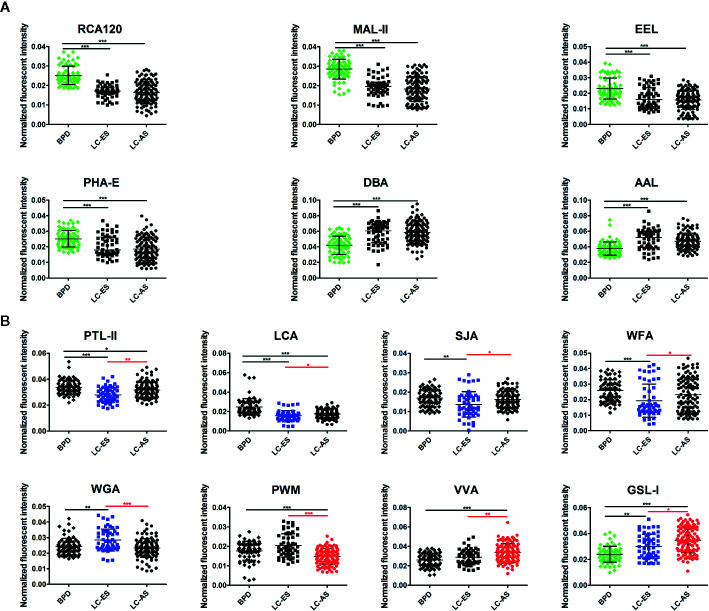
Scatter plot analysis of the data obtained with the 14 candidate lectins first by Kruskal-Wallis test, followed by a Dunn’s Multiple Comparison Test. **(A)** The difference of bronchoalveolar lavage fluid (BALF) glycopatterns between benign pulmonary diseases (BPD) and LC (including different stages) marked with black. **(B)** The difference between early stage lung cancer (LC-ES) and advanced stage lung cancer (LC-AS) marked with red. Those differences was indicated by the p-value (*p < 0.05, **p < 0.01, and ***p < 0.001).

In our study, we hoped to identify which glycans emerged and how the glycans were differentially expressed in BALF during the development of lung cancer. Therefore, 162 patients with a definite clinical stage of 208 lung cancer subjects were further divided into early stage (including stage I/II NSCLC and limited stage SCLC) lung cancer (LC-ES) and advanced stage (including stage III/IV NSCLC and extensive stage SCLC) lung cancer (LC-AS). Similarly, analyses of aberrant glycosylation among BPD, LC-ES, and LC-AS were performed by lectin microarrays. The fluorescent images are displayed in **Figure 3A**. The generated data from three biological replicates of each sample were executed by HCA using Expander 8.0 software to achieve the hierarchical relationship based on the similarities and differences among all glycopattern abundances (**Figure 3B**). The NFIs of each candidate lectin were further represented in a scatter plot. In this differential analysis, 14 lectins revealed significant alterations in BALF glycopatterns among BPD, LC-ES, and LC-AS. As shown in **Figure 4A**, the NFIs of RCA120, MAL-II, EEL, and PHA-E in LC-ES and LC-AS were significantly lower than that of BPD (all *p* < 0.001). In contrast, both DBA and AAL exhibited significantly increased in all the stages of lung cancer compared with BPD (all *p* < 0.001). However, the NFIs of these lectins were not significantly different between LC-ES and LC-AS. As shown in **Figure 4B**, the Gal binders PTL-II, the α-D-Man binders LCA, and the αGalNAc binders SJA as well as the termination in GalNAcα/β1-3/6Gal binders WFA exhibited a decreased in NFIs in patients with LC-ES compared with BPD and LC-AS (all *p* < 0.05). In contrast, an increase in the NFIs of the WGA was observed in patients with LC-ES compared with BPD and LC-AS (all *p* < 0.01). Also, the VVA and the branched (LacNAc)n binders PWM showed significantly decreased or increased NFIs in patients with LC-AS compared with BPD and LC-ES (all *p* < 0.01). In addition, with the development of lung cancer, the NFIs of the αGalNAc binders GSL-I showed a gradual increasing trend from BPD to LC-AS (all *p* < 0.05).

### Validation of the Differential Expression Levels in the BALF Glycopatterns During the Development and Progression of Lung Cancer by SDS-PAGE and Lectin Blotting Analysis

To further validate the different abundances of glycoproteins in BALF from BPD, LC-ES, and LC-AS subjects, SDS-PAGE and lectin blotting analysis were performed with silver staining, Cy5-labeled RCA120, AAL, LCA, and WFA staining, respectively. The results of SDS-PAGE showed similar molecular weights (MWs) and global abundances of BALF proteins for patients with BPD, LC-ES, and LC-AS, except for two apparent different bands with a MW of approximately 25 kDa and 20 kDa, as compared with those of LC-ES (**Figure 3C**). As shown in **Figures 3D, E**, the RCA120 staining displayed a decreased binding tendency from BPD, and LC-ES to LC-AS subjects according to three apparent bands (a1-a3) ranging from 85 to 45 kDa, while the AAL staining displayed stronger binding to three apparent bands (b1-b3) with MWs of approximately 60 kDa, 50 kDa and 40 kDa in LC-AS than that in BPD and LC-ES subjects. LCA staining showed a weaker binding to two apparent bands (c1-c2) with MWs of approximately 60 to 42 kDa in LC-ES and LC-AS than that in BPD subjects. Notably, WFA staining displayed weaker binding to three apparent bands (red arrows) with MWs of approximately 50 kDa, 25 kDa, and 15 kDa in LC-ES than that in BPD and LC-AS subjects. These results were generally consistent with the results from the lectin microarrays.

### Construction of the Diagnostic Models in the Training Cohort Based on BALF Glycopattern Abundances

**Figure 5:**
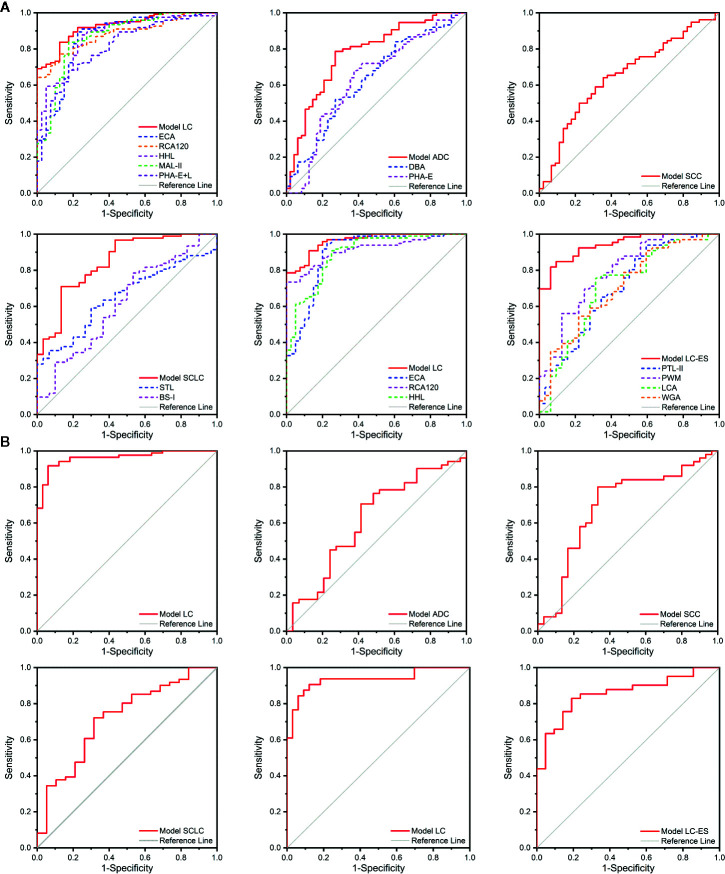
The diagnosis accuracy of the diagnostic models and selected lectins analyzed by receiver operating characteristic (ROC) analysis. **(A)** The ROC analysis for the diagnostic models and candidate lectins in the training cohort. **(B)** The ROC analysis for the diagnostic models in the validation cohort.

The BALF glycopatterns of BPD, ADC, SCC, SCLC, LC-ES, and LC-AS subjects were assessed based on the above lectin microarray data with different types and stages of lung cancer. The detailed information of the ROC analysis for the constructive models and candidate lectins in the training cohort is shown in **Figure 5A** and **Table 2**.

**Table 2:** Detailed information of the receiver operating characteristic (ROC) analysis for the constructive models and candidate lectins in the training cohort and validation cohort.

	AUC	95% confidence interval	Sig.	Cutoff value	Sensitivity	Specificity
**Training cohort**						
**Diagnosis of different types of LC**						
Model LC	0.926	0.886–0.965	<0.001	0.663	0.837	0.875
ECA	0.859	0.787–0.931	<0.001	0.018	0.894	0.775
RCA120	0.881	0.830–0.931	<0.001	0.021	0.772	0.875
HHL	0.870	0.809–0.931	<0.001	0.023	0.878	0.750
MAL-II	0.866	0.798–0.934	<0.001	0.025	0.837	0.825
PHA-E+L	0.810	0.737–0.834	<0.001	0.061	0.683	0.825
Model ADC	0.776	0.690–0.862	<0.001	0.544	0.787	0.729
DBA	0.634	0.532–0.735	0.013	0.052	0.520	0.729
PHA-E	0.634	0.529–0.738	0.013	0.016	0.720	0.583
Model SCC	0.649	0.548–0.749	0.006	0.614	0.641	0.644
Model SCLC	0.846	0.765–0.926	<0.001	0.804	0.710	0.867
STL	0.652	0.552–0.752	0.013	0.019	0.591	0.700
BS-I	0.618	0.501–0.735	0.052	0.018	0.785	0.467
**Diagnosis of different stages of LC**						
Model LC	0.962	0.935–0.989	<0.001	0.666	0.908	0.875
ECA	0.893	0.827–0.959	<0.001	0.018	0.949	0.775
RCA120	0.915	0.833–0.951	<0.001	0.019	0.735	1.000
HHL	0.892	0.870–0.960	<0.001	0.023	0.908	0.750
Model LC-ES	0.941	0.899–0.983	<0.001	0.753	0.818	0.937
PTL-II	0.702	0.587–0.816	0.001	0.025	0.939	0.406
PWM	0.791	0.694–0.888	<0.001	0.017	0.697	0.750
LCA	0.708	0.593–0.823	0.001	0.016	0.742	0.687
WGA	0.709	0.599–0.819	0.001	0.031	0.909	0.406
**Validation cohort**						
**Diagnosis of different types of LC**						
Model LC	0.961	0.929–0.994	<0.001	0.754	0.918	0.939
Model ADC	0.619	0.488–0.750	0.079	0.569	0.706	0.586
Model SCC	0.693	0.567–0.820	0.004	0.578	0.800	0.667
Model SCLC	0.718	0.585–0.851	0.004	0.728	0.721	0.684
**Diagnosis of different stages of LC**						
Model LC	0.935	0.885–0.984	<0.001	0.658	0.906	0.879
Model LC-ES	0.856	0.762–0.950	<0.001	0.668	0.829	0.810

First, the Model LC mathematic formula was established to distinguish lung cancer from BPD using binary logistic regression analysis.

$$Model\;LC=\frac{1}{1+e^{-\left(8.532-293.889*\left(ECA\right)+101.251*\left(GSL-I\right)-275.104*\left(RCA120\right)\right)}}$$

The diagnosis accuracy of Model LC that referred to three lectins (ECA, GSL-I, and RCA120) in the training cohort was appraised by ROC analysis. When judging the types of lung cancer, the results indicated that Model LC had higher discriminatory power for differentiating lung cancer from BPD (AUC: 0.926, sensitivity: 0.837, and specificity: 0.875) than that of these single candidate lectins, such as ECA (AUC: 0.859, sensitivity: 0.894, and specificity: 0.775), RCA120 (AUC: 0.881, sensitivity: 0.772, and specificity: 0.875), HHL (AUC: 0.870, sensitivity: 0.878, and specificity: 0.750), MAL-II (AUC: 0.866, sensitivity: 0.837, and specificity: 0.825), and PHA-E+L (AUC: 0.810, sensitivity: 0.683, and specificity: 0.825). Simultaneously, the ROC curve indicated that Model LC had higher discriminatory power for differentiating different stages of lung cancer from BPD (AUC: 0.962, sensitivity: 0.908, and specificity: 0.875) than that of three candidate lectins, such as ECA (AUC: 0.893, sensitivity: 0.949, and specificity: 0.775), RCA120 (AUC: 0.915, sensitivity: 0.735, and specificity: 1.000), and HHL (AUC: 0.892, sensitivity: 0.908, and specificity: 0.750).

Then, diagnostic models for identifying different types of lung cancer were constructed separately. The Model ADC mathematic formula was built to distinguish ADC from SCC and SCLC using binary logistic regression analysis.

$$Model\;ADC=\frac{1}{1+e^{-\left(4.343-75.290*\left(DBA\right)+111.397*\left(STL\right)-218.004*\left(UEA-I\right)+43.852*\left(BPL\right)\right)}}$$

The diagnosis accuracy of Model ADC that referred to four lectins (DBA, STL, UEA-I, and BPL) in the training cohort was appraised by ROC analysis, indicating that Model ADC had higher discriminatory power for differentiating ADC from SCC and SCLC (AUC: 0.776, sensitivity: 0.787, and specificity: 0.729) than that of the two single candidate lectins, such as DBA (AUC: 0.634, sensitivity: 0.520, and specificity: 0.729), and PHA-E (AUC: 0.634, sensitivity: 0.720, and specificity: 0.583).

The Model SCC mathematic formula was established to diagnose SCC from other subtypes of lung cancer using binary logistic regression analysis.

$$Model\;SCC=\frac{1}{1+e^{-\left(-0.867+34.580*\left(PNA\right)\right)}}$$

The diagnostic accuracy of Model SCC that referred to lectin PNA in the test cohort was appraised by ROC analysis, which resulted in an AUC of 0.649 with a sensitivity of 0.641 and a specificity of 0.644.

The Model SCLC mathematic formula was constructed to differentiate SCLC from NSCLC using binary logistic regression analysis.

$$Model\;SCLC=\frac{1}{1+e^{-\left(1.203-218.357*\left(STL\right)-307.470*\left(BS-I\right)+114.452*\left(PTL-II\right)-413.348*\left(SBA\right)+547.773*\left(PSA\right)+224.736*\left(GNA\right)\right)}}$$

The diagnostic accuracy of Model SCLC that referred to six lectins (STL, BS-I, PTL-II, SBA, PSA, and GNA) in the training cohort was appraised by ROC analysis, indicating that Model SCLC had higher discriminatory power for differentiating SCLC from NSCLC (AUC: 0.846, sensitivity: 0.710, and specificity: 0.867) than two single candidate lectins, such as STL (AUC: 0.652, sensitivity: 0.591, and specificity: 0.700) and BS-I (AUC: 0.618, sensitivity: 0.785, and specificity: 0.467).

Furthermore, a diagnostic model for distinguishing lung cancer patients at different stages was also constructed by binary logistic regression analysis.

$$Model\;LC-ES=\frac{1}{1+e^{-\left(-12.288-360.814*\left(MAL-II\right)+425.102*\left(LTL\right)+195.881*\left(GSL-I\right)+437.281*\left(RCA120\right)+413.840*\left(PTL-II\right)-601.172*\left(PWM\right)\right)}}$$

The diagnostic accuracy of Model LC-ES that referred to six lectins (MAL-II, LTL, GSL-I, RCA120, PTL-II, and PWM) in the training cohort was appraised by ROC analysis, indicating that Model LC-ES also had higher discriminatory power for differentiating LC-ES from LC-AS (AUC: 0.941, sensitivity: 0.818, and specificity: 0.937) than four single candidate lectins, such as PTL-II (AUC: 0.702, sensitivity: 0.939, and specificity: 0.406), PWM (AUC: 0.791, sensitivity: 0.697, and specificity: 0.750), LCA (AUC: 0.708, sensitivity: 0.742, and specificity: 0.687) and WGA (AUC: 0.709, sensitivity: 0.909, and specificity: 0.406).

### Verification of the Diagnostic Models in the Validation Cohort

In order to assess the discriminatory efficiencies, the diagnostic models constructed in the training cohort were then applied to a validation cohort including BPD (n = 33), ADC (n = 32), SCC (n = 32), SCLC (n = 21), LC-ES (n = 22), and LC-AS (n = 42). All subjects preliminarily judged the properties of diseases they underwent through Model LC, and then those patients who were diagnosed with lung cancer by Model LC were further judged using other constructed models to determine their pathological types and stages of cancer. Similarly, ROC analyses were carried out to evaluate the diagnostic accuracy of the constructive models. The detailed results are shown in **Figure 5B** and **Table 2**.

When judging the types of lung cancer, first, the ROC curve showed that Model LC (cutoff value: 0.754, AUC: 0.961, sensitivity: 0.918, and specificity: 0.939) had superior diagnostic accuracy in distinguishing lung cancer from BPD, and it could correctly classify 78 of 85 lung cancer cases and 31 of 33 BPD cases. Next, the ROC curve showed that Model ADC (cutoff value: 0.569, AUC: 0.619, sensitivity: 0.706, and specificity: 0.586) had high diagnostic accuracy in distinguishing ADC from other subjects, and it could correctly classify 17 of 29 ADC cases and 34 of 49 SCC and SCLC cases. The ROC curve showed that Model SCC (cutoff value: 0.578, AUC: 0.693, sensitivity: 0.800, and specificity: 0.667) had high diagnostic accuracy in distinguishing SCC from other subjects, and it could correctly classify 20 of 30 SCC and 38 of 48 ADC and SCLC cases. The ROC curve showed that Model SCLC (cutoff value: 0.728, AUC: 0.718, sensitivity: 0.721, and specificity: 0.684) had high diagnostic accuracy in distinguishing SCLC from other subjects, and it could correctly classify 13 of 19 SCLC cases and 42 of 59 ADC and SCC cases. Meanwhile, when judging the stages of lung cancer, the ROC curve showed that Model LC (cutoff value: 0.658, AUC: 0.935, sensitivity: 0.906, and specificity: 0.879) had superior diagnostic accuracy in distinguishing lung cancer from BPD, and it could correctly classify 58 of 64 lung cancer cases and 29 of 33 BPD cases. Notably, the ROC curve indicated that Model LC-ES (cutoff value: 0.668, AUC: 0.856, sensitivity: 0.829, and specificity: 0.810) had superior diagnostic accuracy in distinguishing LC-ES from other subjects, and it could correctly classify 17 of 21 LC-ES cases and 31 of 37 LC-AS cases.

### Evaluation of the Clinical Application Potential of the Diagnostic Models in the Double-Blind Cohort

Another independent test was developed in a double-blind cohort with 120 subjects to further appraise the clinical application potential of these diagnostic models for differential diagnosis between lung cancer at different types and stages and BPD. A comparison of the double-blind test results and clinical final diagnosis is shown in **Table 3**. In terms of judging the types of lung cancer, 73 of 120 cases of BALF specimens were identified as lung cancer-positive by Model LC. In fact, 72 of 84 lung cancer cases (including 28 of 31 ADC cases, 25 of 32 SCC cases, and 19 of 21 SCLC cases) and 35 of 36 BPD cases were correctly classified by Model LC with an accuracy of 0.892 (107/120). Forty-two of 73 cases of BALF specimens were identified as ADC-positive by Model ADC. In fact, 11 of 28 ADC cases and 41 of 44 other types of lung cancer cases (including 23 of 25 SCC cases and 18 of 19 SCLC cases) were correctly classified by Model ADC with an accuracy of 0.712 (52/73). 34 of 73 cases of BALF specimens were identified as SCC-positive by Model SCC. In fact, 15 of 25 SCC cases and 34 of 47 other types of lung cancer cases (including 23 of 28 ADC cases and 11 of 19 SCLC cases) were correctly classified by Model SCC with an accuracy of 0.671 (49/73). 49 of 73 cases of BALF specimens were identified as SCLC- positive by Model SCLC. In fact, eight of 19 SCLC cases and 49 of 53 other types of lung cancer cases (including 26 of 28 ADC cases and 23 of 25 SCC cases) were correctly classified by Model SCLC with an accuracy of 0.787 (57/73). Notably, when judging the stages of lung cancer, 66 of 109 cases of BALF specimens were identified as LC-positive by Model LC. In fact, 65 of 73 lung cancer cases (including 22 of 27 LC-ES cases and 43 of 46 LC-AS cases) and 35 of 36 BPD cases were correctly classified by Model LC with an accuracy of 0.917 (100/109). Forty of 66 cases of BALF samples were identified as LC-ES-positive by Model LC-ES. In fact, 17 of 22 LC-ES cases and 40 of 43 LC-AS cases were correctly classified by Model LC-ES with an accuracy of 0.864 (57/66). 

**Table 3:** The results of the double-blind test compared with clinical final diagnosis.

Clinical final diagnosis^a^	Diagnostic models	False-positive	False-negative	Sensitivity	Specificity	Accuracy
BPD (36)	Model LC (47)	1	12	0.856 (72/84)	0.972 (35/36)	0.892 (107/120)
ADC (31)	Model ADC (14)	17	3	0.932 (41/44)	0.393 (11/28)	0.712 (52/73)
SCC (32)	Model SCC (28)	10	13	0.723 (34/47)	0.600 (15/25)	0.671 (49/73)
SCLC (21)	Model SCLC (12)	11	4	0.925 (49/53)	0.421 (8/19)	0.781 (57/73)
LC-ES&LC-AS (73)	Model LC (66)	1	8	0.890 (65/73)	0.972 (35/36)	0.917 (100/109)
LC-ES (27)	Model LC-ES (20)	5	3	0.930 (40/43)	0.773 (17/22)	0.864 (57/66)
LC-AS (46)	Model LC-ES (45)					

^a^Detailed clinicopathological characteristics of 120 patients in the double-blind cohort were shown in **Table 1**. BPD, benign pulmonary disease; ADC, adenocarcinoma; SCC, squamous carcinoma; SCLC, small cell lung cancer; LC-ES, early stage lung cancer; LC-AS, advanced stage lung cancer.

## Discussion

Lung cancer is a severe health problem that prevails around the world (43). At present, fiberoptic bronchoscopy is a standard procedure of the diagnostic work-up of patients with suspected respiratory system lesions to obtain specimens for histological or cytological examination. Naturally, BALF is sampled before biopsy during this process, gathering specimens that are difficult to detect by bronchoscopy. However, the cytological examination for BALF is greatly influenced by artificial factors and its diagnostic accuracy is relatively poor. Proteins are the major component of BALF and have been recognized to play valuable roles in the discovery of biomarkers for lung cancer. Comparative analyses have documented that certain proteins are present at higher levels in BALF than in plasma, suggesting that they are specifically produced in the respiratory tract (44, 45). Protein glycosylation is the enzymatic addition of sugars or oligosaccharides to proteins, which leads to the functional diversity of proteins and participates in the diversity of their biological activities, particularly in cancer genesis and progression (46). Due to the complexity of glycan structures and the heterogeneity of glycosylation sites, it is a challenge for the complete characterization of tumor glycomics and glycoproteomics represents (47, 48). To date, aberrant glycosylation has been observed in patients with various types of cancer, such as gastric, breast, and colorectal cancer (25, 49, 50). In recent years, research on the changes in glycosylation in lung cancer has made extensive progress, providing a new thread for its diagnosis and therapy (23). Lectin microarray as a high-throughput analytical glycoscience strategy allows rapid observation of different glycans following minimal sample preparation, which guarantees the real state of protein glycosylation in clinical samples from body fluids of health, benign lesion, and cancer being accurately reflected (35). Moreover, lectins, which bind to the glycan of the glycoproteins, can be exploited to identify abnormal glycopatterns, which in turn would contribute to increasing the specificity of cancer diagnosis.

In our study, first, the alterations in glycoproteins in 281 individual BALF subjects were systematically probed by lectin microarrays and lectin blotting analysis; then, those participants were randomly assigned into a training cohort and validation cohort for the construction and verification of diagnostic models, respectively. Moreover, an additional 120 newly collected BALF samples enrolled in the double-blind cohort were independently detected to examine the diagnostic accuracy of the diagnostic models. According to the research results, there were 15 lectins (e.g., PHA-E, EEL, and BPL) that contribute to significant alterations in the BALF glycopatterns among BPD, ADC, SCC and SCLC through statistical analysis. Meanwhile, 14 lectins (e.g., PTL-II, LCA, and SJA) revealed noticeable alterations in the BALF glycopatterns between the control group and lung cancer patients at different stages, and the validation results of lectin blotting were generally consistent with the results from lectin microarrays. The findings indicated that the expression levels of Tn antigen and its derived structure T antigen recognized by GSL-I, VVA, and DBA in BALF were up-regulated both in different subtypes and stages of lung cancer compared with BPD, of which the level of VVA was significantly higher in ADC than that in BPD and SCC. Tn antigen is one of the most specific tumor-associated carbohydrate structures that is not normally expressed in peripheral tissues or blood cells but can promote tumor cell invasion (51). The expression of this antigen found in most human carcinomas is derived from blockade during the normal O-glycosylation pathway, in which glycans extend from the common precursor GalNAcα1-O-Ser/Thr (Tn antigen) (52). Similar to our results, an earlier study found that T and Tn antigen in ADC were detected at a higher frequency than in SCC by immunohistochemical staining (53). Moreover, the present study also observed that the expression levels of GSL-I and VVA gradually increased with the stages of lung cancer, which may reflect the tumor burden and is related to the poor prognosis of pulmonary disease. Our previous study also demonstrated that the level of T antigen in serum was increased in patients with stage III and stage IV ADC compared with levels in healthy controls (40). Based on the above results, the differential expression levels of T and Tn antigen may have potential as biomarkers that not only recognize lung cancer and distinguish the histological subtypes of NSCLC but also may serve as a prognostic indicator for lung cancer.

In mammals, core fucosylation, a typical terminal modification of proteins, is the addition of α1-6-linked fucose to the innermost GlcNAc residue of N-glycans, which is only catalyzed by fucosyltransferase 8 (FUT8) (54). Studies frequently reported that FUT8 is highly expressed in many malignant diseases, such as lung, breast, and colorectal carcinomas, but it is negatively correlated with the development of gastric cancer (55–58). Hirao et al. (42) performed lectin microarray analysis of lung cancer tissues and cell lines and identified AAL as a lectin probe specific to NSCLC. In line with these findings mentioned above, the expression level of core-fucosylation recognized by AAL was elevated in different types and stages of lung cancer compared with expression in BPD in this study. Of particular relevance for our research, the binding performance of Gal and GalNAc glycans recognized by BPL, PNA, MPL, SJA, WFA, WGA, and PWM deserves our attention, which may help with pathological typing and early diagnosis of lung cancer. Among these lectins, compared with SCC, the expression of BPL decreased significantly in ADC. The expression of PNA and MPL decreased significantly in SCC compared with expression in ADC and SCLC. Simultaneously, the expression of SJA and WFA related to LC-ES was down-regulated in comparison with that in LC-AS, however, the expression of WGA and PWM was up-regulated in LC-ES, and showed a same expression trend as BPD. This finding reminds us the glycans recognized by these four lectins provide important information for the early diagnosis of lung cancer. In addition, the expression levels of Gal and GalNAc structures recognized by RCA120, EEL, ECA, PTL-II, and PHA-E, as well as sialylated structure binders MAL-II were significantly decreased in different types and stages of lung cancer, while the expression levels of DSA and GSL-II increased significantly in lung cancer compared with levels in BPD, especially in ADC. To sum up, according to the research results, these lectins (MAL-II, RCA120, ECA, HHL, DBA, DSA, and AAL) have potential to become biomarkers for the diagnosis of lung cancer. Moreover, ADC and SCC may be distinguished by PHA-E, EEL, BPL, GSL-II, and VVA. Also, PNA and MPL can be used to distinguish SCC and SCLC. Notably, PTL-II, LCA, SJA, WFA, WGA, and PWM are expected to be valuable biomarkers for the early diagnosis of lung cancer.

Furthermore, we constructed five diagnostic models (Model LC, Model ADC, Model SCC, Model SCLC, and Model LC-ES) based on BALF glycopattern abundances for the differential diagnosis between benign and malignant lung diseases, as well as the classification and periodization of lung cancer. The distinguishing performance of all models was better than that of single lectins. Model LC, Model SCLC, and Model LC-ES achieved desired diagnostic powers with an AUC value greater than 0.700 (*p <* 0.01) for the diagnosis of lung cancer, SCLC, and LC-ES in both the test and validation cohorts. In addition, Model LC and Model LC-ES exhibited high accuracies of 0.917 and 0.864 in the double-blind cohort, respectively, which are clinically valuable for the identification of benign and malignant pulmonary diseases and early diagnosis of lung cancer with stable and reliable BALF glycopattern biomarkers. However, the sample discrimination abilities of Model ADC, Model SCC, and Model SCLC are not as good as the above two diagnostic models. Therefore, the subtle differences in glycosylation hidden behind different pathological types of lung cancer need to be further explored.

There are still some limitations in this study. One is that the clinical sample size of patients with LC-ES is relatively small, and the other is that our research has not involved the detailed molecular mechanism that causes aberrant glycosylation for the progression of lung cancer. Further investigations in larger cohorts are required to assess the clinical application potential of these BALF glycopattern biomarkers in diagnosing lung cancer and even distinguishing other cancers in the future. We will also focus on the glycosylated pathway related to the development of lung cancer and intend to elucidate the correlation between abnormal glycosylation alterations and malignant biological behaviors.

In conclusion, our current study systematically explored the lung cancer-related changes in BALF glycosylation and detected differentially expressed glycoproteins among patients with BPD, ADC, SCC, SCLC, LC-ES, and LC-AS by lectin microarrays and blotting analysis, which indicated that different combinations of lectins can be used to detect the type of lung cancer, and even its pathological stage. Further, five diagnostic m**o**dels with better discrimination were constructed to distinguish different types and stages of lung cancer, and Model LC and Model LC-ES revealed high accuracy greater than 0.850 in the double-blind test, which may contribute to identifying benign and malignant pulmonary diseases and diagnosing lung cancer at an early stage. This study provides insight into the discovery of promising biomarkers for the diagnosis of lung cancer based primarily on the precision alterations in BALF glycopatterns.

## Data Availability Statement

The raw data supporting the conclusions of this article will be made available by the authors, without undue reservation.

## Ethics Statement

The studies involving human participants were reviewed and approved by the Ethical Committee of the First Affiliated Hospital of Xi’an Jiao Tong University in Xi’an, China. The patients/participants provided their written informed consent to participate in this study.

## Author Contributions

XG, ZL, MC, and LL conceived and designed experiments. LL, DL, LW, and TC contributed to collecting clinical specimens. LL, JS, FZ, and CZ performed experiments. LL, DL, JS, FZ, HY, and CZ analyzed and interpreted data. LL and DL wrote the manuscript with help from XG, ZL, MC, and JS for revision. All authors contributed to the article and approved the submitted version.

## Funding

This work is supported by the Key Research and Development Program of Shaanxi Province (Grant No. 2019SF-215) and the National Natural Science Foundation of China (Grant No. 81871955).

## Conflict of Interest

The authors declare that the research was conducted in the absence of any commercial or financial relationships that could be construed as a potential conflict of interest.
