# Supplementary material for: Protein Glycopatterns in Bronchoalveolar Lavage Fluid as Novel Potential Biomarkers for Diagnosis of Lung Cancer
Source: Front Oncol. 2021 Jan 14;10:568433. doi: 10.3389/fonc.2020.568433 (PMC7840895; doi:10.3389/fonc.2020.568433)
Supplement: Supplementary file 1 [file DataSheet_1.pdf]

Supplementary Material

Table S1. The glycan specificities of 37 lectins.

Lectin	Specificity
Jacalin	Galβ1-3GalNAcα-Ser/Thr(T), GalNAcα-Ser/Thr(Tn), GlcNAcβ1-3-GalNAcα-Ser/Thr(Core3), sialyl-T(ST). not bind to Core2, Core6, and sialyl-Tn (STn)
ECA	Galβ-1,4GlcNAc (type II), Galβ1-3GlcNAc (type I)
HHL	High-Mannose, Manα1-3Man, Manα1-6Man, Man5-GlcNAc2-Asn
WFA	terminating in GalNAcα/β1-3/6Gal
GSL-II	GlcNAc and agalactosylated tri/tetra antennary glycans
MAL-II	Siaα2-3Galβ1-4Glc(NAc)/Glc, Siaα2-3Gal, Siaα2-3, Siaα2-3GalNAc
PHA-E	Bisecting GlcNAc, biantennary complex-type N-glycan with outer Gal
PTL-I	GalNAc, GalNAcα-1,3Gal, GalNAcα-1,3Galβ-1,3/4Glc
SJA	αGalNAc, αGal, anti-A and BTerminal in GalNAc and Gal, anti-A and anti-B human blood group
PNA	Galβ1-3GalNAcα-Ser/Thr(T)
EEL	Galα1-3(Fucα1-2)Gal (blood group B antigen)
AAL	Fucα1-6 GlcNAc(core fucose), Fucα1-3(Galβ1-4)GlcNAc
LTL	Fucα1-2Galβ1-4GlcNAc, Fucα1-3(Galβ1-4)GlcNAc, anti-H blood group specificity
MPL	Galβ1-3GalNAc, GalNAc
LEL	(GlcNAc)n, high mannose-type N-glycans
GSL-I	αGalNAc, αGal, anti-A and B
DBA	αGalNAc, Tn antigen, GalNAcα1-3((Fucα1-2))Gal (blood group A antigen)
LCA	α-D-Man, Fucα-1,6GlcNAc, α-D-Glc
RCA120	β-Gal, Galβ-1,4GlcNAc (type II), Galβ1-3GlcNAc (type I)
STL	trimers and tetramers of GlcNAc, core (GlcNAc) of N-glycan, oligosaccharide containing GlcNAc and MurNAc
BS-I	α-Gal, α-GalNAc, Galα-1,3Gal, Galα-1,6Glc
ConA	High-Mannose, Manα1-6(Manα1-3)Man, terminal GlcNAc
PTL-II	Gal, blood group H, T-antigen
DSA	β-D-GlcNA, (GlcNAcβ1-4)n, Galβ1-4GlcNAc
SBA	α- or β-linked terminal GalNAc, (GalNAc)n, GalNAcα1-3Gal, blood-group A antigen
VVA	terminal GalNAc, GalNAcα-Ser/Thr(Tn), GalNAcα1-3Gal
NPA	High-Mannose, Manα1-6Man
PSA	α-D-Man, Fucα-1,6GlcNAc, α-D-Glc
ACA	Galβ1-3GalNAcα-Ser/Thr (T antigen), sialyl-T(ST) tissue staining patterns are markedly different than those obtained with either PNA or Jacalin
WGA	Multivalent Sia and (GlcNAc)n
UEA-I	Fucα1-2Galβ1-4Glc(NAc)
PWM	Branched (LacNAc)n
MAL-I	Galβ-1,4GlcNAc, Siaα2-3Gal, Galβ1-3GlcNAc, Siaα2-3
GNA	High-Mannose, Manα1-3Man
BPL	Galβ1-3GalNAc, Terminal GalNAc
PHA-E+L	Bisecting GlcNAc, bi-antennary N-glycans, tri- and tetra-antennary complex-type N-glycan
SNA	Sia2-6Gal/GalNAc
